# Preference Dynamics in Sequential Consumer Choice with Defaults

**DOI:** 10.1177/0022243720956642

**Published:** 2020-10-14

**Authors:** Bas Donkers, Benedict G.C. Dellaert, Rory M. Waisman, Gerald Häubl

**Keywords:** behavioral decision theory, choice spillover, defaults, field study, preference construction, product customization, sequential consumer choice

## Abstract

This research examines the impact of defaults on product choice in sequential-decision settings. Whereas prior research has shown that a default can affect what consumers purchase by promoting choice of the preselected option, the influence of defaults is more nuanced when consumers make a series of related choices. In such a setting, consumer preferences may evolve across choices due to “spillover” effects from one choice to subsequent choices. The authors hypothesize that defaults systematically attenuate choice spillover effects because accepting a default is a more passive process than either choosing a nondefault option in the presence of a default or making a choice in the absence of a default. Three experiments and a field study provide compelling evidence for such default-induced changes in choice spillover effects. The findings show that firms’ setting of high-price defaults with the aim of influencing consumers to choose more expensive products can backfire through the attenuation of spillover. In addition to advancing the understanding of the interplay between defaults and preference dynamics, insights from this research have important practical implications for firms applying defaults in sequential choices.

Firms that aim to sell products to consumers inevitably are “choice architects” in that they must decide how to present their product offerings to prospective buyers (Johnson et al. 2012; Thaler and Sunstein 2008). Setting a default—that is, prespecifying an option that automatically becomes the selected one unless a consumer actively selects another available option—is an important tool of choice architecture. Defaults have been shown to have a powerful impact on consumers’ decisions (Brown and Krishna 2004; Johnson and Goldstein 2003; Madrian and Shea 2001; Smith, Goldstein, and Johnson 2013; Steffel, Williams, and Pogacar 2016; Sunstein 2013).

Default effects have been examined extensively in settings where consumers make a single choice. However, little is known about the impact of defaults when consumers make a sequence of related choices. This is the case, for example, when products are presented in a format that allows consumers to configure their product by selecting the specific options they desire for each of a number of product modules (e.g., when configuring automobiles, pieces of furniture, holiday packages, or financial services) or when consumers make several choices in connection with an overall consumption experience (e.g., booking a flight, hotel, and rental car for a trip; configuring a multicourse meal at a restaurant). Such complex decisions are best thought of as sequential choice processes because consumers typically make them by selecting one option at a time.

Selecting one of the available options for each of a sequence of related choices can be an onerous task (Dellaert and Stremersch 2005; Hildebrand, Häubl, and Herrmann 2014). Therefore, presenting consumers with defaults in the form of preselected options has the potential to be helpful in that it may simplify the choice process. Typically, making the choice process less cumbersome for consumers is also in the interest of the firm (e.g., by reducing the risk that the process will not result in a purchase). In addition, firms can use defaults in an attempt to deliberately influence consumers’ choices in a particular direction—for instance, to promote the sale of products that are more profitable or to guide consumers toward products that might render them more satisfied. Firms typically have many degrees of freedom in setting defaults in a sequence of choices. For each individual choice, the firm can control whether one of the options is preselected as a default and, if so, which specific option is set as the default.

Prior work has shown that setting an option as a default renders it more likely to be chosen (Johnson and Goldstein 2003; Sunstein 2013). However, whether and how setting a default for a particular choice influences what options consumers select in *subsequent* choices has not been examined to date. Evidence from research on sequences of choices (in the absence of any defaults) suggests that the choices a consumer makes can have systematic “spillover” effects on their subsequent choices (Levav et al. 2010; Priester, Dholakia, and Fleming 2004; Simon et al. 2008; Simonson and Tversky 1992; Wilcox and Song 2011). The current work examines how defaults moderate such choice spillover.

We focus on how defaults affect choice spillover that is inference-based in that it results from what consumers infer, either about their own preferences or about the market environment, from the choices they make. Our key hypothesis is that inference-based choice spillover depends on whether the initial choice is the result of the consumer accepting a default versus selecting a nondefault option. Choice spillover may differ depending on whether choices are made actively versus more passively (Greenberg and Spiller 2016; Schrift and Parker 2014). Selection of a default option does not require extensive deliberation, as the consumer can simply accept the default, so it represents a comparatively less thoughtful act than selecting the same option when it is not the default. Therefore, we predict that the more passive choice of merely accepting a default attenuates inference-based choice spillover from such a choice to subsequent choices.

In the next section, we outline our theorizing about the nature of preference dynamics in sequential choice processes and about the role of defaults in these dynamics. Then, we present evidence from three experiments that were designed to test the key predictions arising from this theory. This is followed by an examination of the economic consequences of multiple defaults in a field study conducted with a major automobile manufacturer in which consumers configured their cars using the manufacturer’s online product customization system. Converging evidence from these four studies provides clear support for our theorizing—defaults not only have a direct impact on the choices for which they are set but (if accepted) also attenuate inference-based choice spillover effects on subsequent choices. The article concludes with a discussion of the theoretical and practical implications of these findings.

## Dynamic Effects of Defaults in Sequential Consumer Choice

We develop our theorizing about the dynamic effects of defaults on consumer choice in three steps. First, we consider the immediate impact of defaults (i.e., how they influence choice when one of the options is preselected). This is followed by a discussion of spillover effects in sequences of choices, whereby consumers’ preferences in connection with a particular choice can be systematically affected by choices they made previously. Finally, we theorize about how defaults moderate spillover effects to subsequent choices by interfering with inference processes that drive choice spillover.

### The Immediate Effects of Defaults

There is ample evidence that defaults affect the choices people make, typically boosting uptake of a default option (Brown and Krishna 2004; Johnson and Goldstein 2003; Madrian and Shea 2001; Sunstein 2013). Various explanations for this immediate effect of defaults have been provided (Brown and Krishna 2004; Dinner et al. 2011). First, consumers may choose the default option to reduce the cognitive effort associated with making a decision (Johnson, Bellman, and Lohse 2002; Thaler and Sunstein 2008). Individuals make trade-offs between the cognitive resources they allocate to a decision and the importance or value of making a good decision (Dickhaut, Rustichini, and Smith 2009; Gershman, Horvitz, and Tenenbaum 2015). Passive acceptance of a default can be an effective way of conserving cognitive resources. In addition, consumers may make assumptions about a firm’s reasons for setting a default and interpret a default as an implicit recommendation to select a particular option (McKenzie, Liersch, and Finkelstein 2006). Thus, consumers might perceive defaults as endorsements by the firm and be inclined to select a default option for that reason. Finally, consumers may feel as though they are missing out on the default if they choose not to select it. Thus, they may be averse to the (perceived) loss of the default if they were to select another option, which can cause them to accept the default (Park, Jun, and MacInnis 2000; Smith, Goldstein, and Johnson 2013).

### Dynamic Effects in Sequential Choice

Consumers tend to construct their preferences, in part, on the basis of the way in which a set of choice options is presented (Bettman, Luce, and Payne 1998; Lichtenstein and Slovic 2006; Payne, Bettman, and Schkade 1999; Tversky, Sattath, and Slovic 1988). Moreover, there is evidence that constructed preferences influence not only consumers’ choices in the particular settings in which they originate but also their subsequent choices (Levav et al. 2010; Simon et al. 2008; Simon, Snow, and Read 2004). Such spillover effects can originate from various underlying sources. We focus on choice spillover effects that are inference-based in that they result from what consumers infer, either about their own preferences or about the market environment, from their choices.

Central to our theorizing is a type of inference-based choice spillover that we label “preference updating.” We conceptualize preference updating as spillover that arises when preferences constructed while making a choice (e.g., preference for a high-quality option) persist to influence a subsequent choice. The literature on self-perception (e.g., Bem 1967) and cognitive dissonance (e.g., Festinger 1962) suggests that individuals tend to display consistency in their preferences across related choices. Moreover, people may infer internal states (e.g., their attitudes or preferences) from their own overt actions, giving rise to an updating of these internal states as a consequence of the choices they make. For instance, the mere act of selecting a high-price option in one choice might signal a general preference for high-quality (and thus expensive) options to the consumer, in turn rendering them more likely to select high-price options in subsequent choices. While the choices a consumer makes reflect, at least in part, their prior preferences, those preferences evolve through an ongoing process of preference updating that is informed by inferences from the evaluation of the presented options and the choices made. Thus, the inferential processes involved in preference construction influence the preferences that spill over from one choice to the next. The result is a positive choice spillover such that the probability that the preference expressed in the subsequent choice will be consistent with the preference exhibited in the current choice is increased.

Another type of inference-based choice spillover is one that arises from consumer inferences about the market. The background-contrast effect (Simonsohn 2006; Simonson and Tversky 1992) is a prominent example of such inference making. It results from the influence of the composition of a set of options from which a consumer has previously made a choice. For instance, one might use the attribute levels of the options in a choice set to make inferences about the trade-off between the attributes in a given domain, such as how large a difference in price is (or should be) associated with a particular difference in product quality. These inferences then serve as the background against which the options that are available in a subsequent choice are evaluated, which produces a spillover. For example, if consumers are able to obtain a large quality improvement for only a very small increase in price in one choice, this affects the inferences they make regarding the relationship between quality and price in that domain (Prelec, Wernerfelt, and Zettelmeyer 1997), and these inferences may spill over to how much more consumers are subsequently willing to pay for higher-quality options in the same domain (Simonson and Tversky 1992)—in this example, less than they might have otherwise. The result is a negative choice spillover such that the probability that the preference expressed in the later choice will be consistent with the preference exhibited in the earlier choice is decreased. (In the current example, the same price–quality trade-off that promotes selection of a higher-quality option in the earlier choice serves as the background that promotes selection of a lower-quality option in the subsequent choice.)

A common form of choice spillover that does not require inference making is balancing, whereby consumers compensate for making choices reflecting one particular preference direction by subsequently shifting their choices in the opposite direction (Dhar and Simonson 1999; Khan and Dhar 2006). Unlike preference updating and background-contrast effects, balancing is primarily driven by the outcome of an earlier choice as it relates to some overall target or goal. For instance, balancing can be the result of consumers considering the outcome of a particular choice in light of an overall target that they have in mind for an entire series of choices, such as a total amount of money to spend or a total number of calories to consume. Thus, balancing may manifest in a pattern of choices where, for example, selecting a high-price option on one occasion renders a consumer more likely to select a low-price option on a subsequent occasion, producing a negative choice spillover.

In summary, choices made earlier in a sequence of related choices may have spillover effects on subsequent choices. Preference updating and background-contrast effects are instances of choice spillover effects that are driven by what consumers infer from their own choices, whereas balancing is a form of choice spillover that results from how the option selected on an earlier occasion affected some overall target or goal. Next, we consider the moderating effect of defaults on choice spillover.

### Dynamic Effects in Sequential Choice with Defaults

Spillover effects across choices may vary as a function of the circumstances under which choices are made (Häubl and Murray 2003; Khan, Zhu, and Kalra 2011; Priester, Dholakia, and Flemming 2004; Yoon and Simonson 2008). We propose that a critical determinant of spillover effects from one choice to another is whether the option that is selected in the earlier choice was set as a default or not.

Spillover effects may depend on whether choices are made actively versus more passively (Greenberg and Spiller 2016; Schrift and Parker 2014). Selection of a default option does not require extensive deliberation, as the consumer can simply accept the default. Consequently, choosing the default option represents a more passive choice than (1) selecting an option other than the default and (2) selecting an option in a setting where none of the options was set as a default. The more passive nature of the process of accepting the default—compared with actively choosing an option that was not the default—implies a less thoughtful decision. This is in line with the finding that individuals who accepted a default subsequently have a stronger desire to switch to a different option (Brown, Farrell, and Weisbenner 2016).

The thoughtfulness of the decision process has important implications both for the decision itself and for how it may influence subsequent decisions. Inference-based choice spillover effects require a thoughtful decision process (Dhar and Gorlin 2013). Prior research has shown that context effects are less pronounced when consumers deliberate less about concrete (Khan, Zhu, and Kalra 2011) and attribute-level (Mantel and Kardes 1999) considerations. For example, background-contrast effects are attenuated when thoughtfulness is compromised (Priester, Dholakia, and Flemming 2004). This is not surprising, because inferences that individuals make about market conditions—through active processing of attribute information—play an important role in background-contrast effects (Prelec, Wernerfelt, and Zettelmeyer 1997). The less thoughtful act of accepting a default should thus reduce background-contrast effects on subsequent choices.

In the case of preference updating, the less thoughtful choice of a default option should make that choice less informative about consumers’ own preferences and, thus, suppress any inferences about their preferences that consumers might make from it, compared with a choice that is made more actively. Therefore, we hypothesize that defaults attenuate preference updating if—and only if—they are accepted, and that they do so as a result of the less thoughtful processing associated with passive default acceptance compared with the active selection of a nondefault option.

In the case of balancing, spillover effects result from pursuit of multiple choice-related goals (Dhar and Simonson 1999), such as the desire to buy a nice car within a particular budget, or wishing to limit calorie intake while enjoying a tasty meal. While the thought process leading to a choice might have a smaller impact on inferences that can influence subsequent choices when the choice process was less active, the impact of the choice on goal achievement need not be affected by the process leading to the choice and thus may not be attenuated by defaults. Therefore, in contrast to our predictions that default acceptance attenuates inference-based choice spillover, balancing effects should be less sensitive to the thought processes involved in making earlier choices. An example of this is the case in which consumers are balancing multiple choices under a budget constraint. Staying within budget when considering a choice requires that consumers account for prior choices in the sequence irrespective of whether the previous choices were made actively or passively. The presence or absence of a default in a prior choice is thus immaterial to such a budget-based balancing process.

Finally, even if consumers choose an option other than the default, the mere presence of a default, and its active rejection, may still affect inference-based choice spillover compared with making the same choice in the absence of a default. First, rejecting a default requires consideration of why the preselected option is not acceptable and may also prompt speculation about the choice architect’s motive in specifying the default. Such deliberations could make rejecting the default an even more thoughtful choice than making the same selection in the absence of a default and, thus, increase choice spillover. Second, the fact that a particular option was set as the default may be interpreted as a recommendation, which could lead to preference updating in the direction of the default option. Such a recommendation effect could counteract, and potentially reverse, the hypothesized choice spillover effect resulting from active rejection of a default.

In summary, the essence of our theorizing is as follows. Choice of a particular option either in the absence of a default or when a default is present but is rejected represents an active choice that supports consumer inference making, thus promoting spillover to subsequent choices. Preference updating results in a positive choice spillover, whereas background contrast effects produce negative choice spillover. If the selection of an option takes the form of default acceptance, representing a more passive choice, consumer inference making is suppressed and, as a result, the preference updating and background contrast effects are attenuated (relative to the same option being chosen actively). Because balancing is less inference-based, it is less sensitive to whether choices are made via default acceptance.

## Experiment 1: Sequential Choice of Similar Products

Experiment 1 aims to test the hypothesis that selecting a particular option represents a less thoughtful, more passive choice when it is a default than when it is not and, consequently, attenuates inference-based spillover effects on a subsequent choice. We employed a paradigm designed to elicit a background-contrast effect (Simonson and Tversky 1992), a well-established form of choice spillover that is sensitive to decision-maker thoughtfulness (Priester, Dholakia, and Flemming 2004).

Background-contrast effects emerge when attribute trade-offs that are manifest in a choice among a set of options differ substantially from those experienced in a previous choice (among different options) within the same domain (Simonson and Tversky 1992). For instance, if the difference in price between a lower- and a higher-quality product (e.g., a three-star and a four-star hotel) in an initial choice is very large (very small), a background is established against which a moderate price difference between these same two quality tiers in a subsequent choice is then perceived to be smaller (larger). The result is a negative spillover effect such that greater inclination to choose a low-quality (high-quality) option in the first choice is followed by greater preference for a high-quality (low-quality) option in the next choice.

Background-contrast effects hinge on decision making being sufficiently thoughtful to support the generation of an inference about attribute trade-offs from the initial choice and application of that inference to the subsequent choice. Indeed, prior work has shown that such effects are suppressed under conditions that engender low thoughtfulness (Priester, Dholakia, and Flemming 2004). If, as we argue, accepting a default is a passive, less thoughtful, choice, the background-contrast effect should be attenuated when the initial selection is made by merely accepting the default option (compared with a more active choice).

### Method

A total of 2,498 residents of the United States recruited from Amazon’s Mechanical Turk platform completed the experiment. After providing basic demographic information (gender, age, and language most commonly spoken), participants were presented with an instructional manipulation check (IMC; Oppenheimer, Meyvis, and Davidenko 2009). Those who failed the IMC were directed to reread the instructions before continuing, and those who failed a second time (138) were dismissed and therefore did not complete the experiment. At the end of the study, participants responded to a distraction self-report,^1^ were shown a debriefing statement, and were asked to either confirm their consent or withdraw their data.

Participants were randomly assigned to one of the five conditions of a 2 (default: absent vs. present) × 2 (background contrast: inexpensive vs. expensive) plus control between-subjects design. They all first completed four training choices, which entailed indicating one’s preference among pairs of paintings, to become familiar with the choice interface and the manner in which defaults were implemented.^2^ Next, participants were instructed to imagine that they were planning a trip to Canada and that they were to choose a hotel in each of two cities—Toronto and Montreal. For each city, participants chose between a higher-quality, higher-price (four-star) and a lower-quality, lower-price (three-star) hotel. In the experimental conditions, participants first selected a Toronto hotel and then a Montreal hotel. The former (Toronto) was used to manipulate the background contrast, whereas the latter (Montreal) served as the focal choice. In the control condition, the focal choice (Montreal) was the first hotel choice participants made, followed by the Toronto hotel choice. This condition enables a quantification of the baseline spillover associated with the different background contrasts.

We conducted a series of pretests to calibrate the stimuli (for all experiments).^3^ Table 1 provides an overview of the hotel stimuli used in Experiment 1. For the focal (Montreal) choice, the prices of the three-star and four-star options were $129 and $159, respectively. The prices of the Toronto hotels were manipulated to induce the desired background-contrast effects with the aim that the option that is favored by a particular contrast would be preferred by the vast majority of participants. In the inexpensive-background-contrast condition, the higher-quality Toronto option is favored because the price difference between the quality tiers is small ($5). Conversely, in the expensive-background-contrast condition, the lower-quality Toronto option is favored due to a large price difference between the quality tiers ($150). In the default-present conditions, the default was applied to the option that is favored by the background contrast. Consequently, most participants were expected to choose this option irrespective of whether a default was set. This design allows us to test the effect of the mere presence of a default in the first (Toronto) choice on subsequent preference as revealed in participants’ focal (Montreal) choice and enables valid comparisons of choice shares among conditions.

**Table 1.** Options for Hotel Choices.

		Low-Price Option	High-Price Option
Toronto choice: inexpensive background contrast	Quality	Three-star standard	Four-star luxury
	Price	$134 per night	$139 per night
Toronto choice: expensive background contrast	Quality	Three-star standard	Four-star luxury
	Price	$119 per night	$269 per night
Montreal choice: focal choice	Quality	Three-star standard	Four-star luxury
	Price	$129 per night	$159 per night

### Results and Discussion

Data from 107 participants were excluded from analysis based on self-reported distractions or technical problems. This leaves a usable sample of 2,391 (M_age_ = 38.22 years, SD_age_ = 12.39 years; 56.4% female).

We fit a logit model to examine the likelihood of choosing the higher-price hotel in the first (Toronto) choice as a function of background contrast, default, and their interaction. Only a main effect of background contrast emerges (b = −4.11, *p* < .001). As we expected, there is no main effect of default in the first choice (b = .05, *p* = .837), nor is the interaction significant (b = −.30, *p* = .323). As we intended, the vast majority of participants in the experimental conditions chose the background-favored option—that is, the lower (higher) quality hotel in the expensive (inexpensive) background-contrast condition (no-default conditions: 85.4% and 91.3%, respectively; default conditions: 88.2% and 91.6%, respectively).

The results of the focal (Montreal) hotel choice (Table 2) reveal a substantial difference in choice shares between the two background-contrast conditions. We fit a logit model to confirm the background-contrast effect and test for attenuation of this effect by the default. We examine the likelihood of choosing the higher-price hotel in the focal (Montreal) choice as a function of background contrast, default, and their interaction. First, choice shares are affected by the background contrast (b = 1.18, *p* < .001) and by the presence of a default (b = .30, *p* = .024). Importantly, these main effects are qualified by a significant interaction (b = −.54, *p* = .004), indicating that the spillover effect is attenuated when the background-favored option has been set as a default in the initial choice. Considering that the vast majority selects this option in the initial choice, irrespective of whether it has been preselected as a default, this interaction supports our hypothesis that choosing an option when it is a default (i.e., making a passive choice) attenuates the choice spillover effect relative to when it is not.

**Table 2.** Choice Share Results (Experiment 1).

	Choice Share of High-Price Option in Focal Choice (Montreal Hotel)	
	Default Absent	Default Present
Inexpensive background contrast	38.2%	45.4%
Expensive background contrast	66.7%	61.0%
Control (no background choice)	45.6%	

Comparing the choice shares in the experimental conditions with those in the control condition, where the focal choice was not preceded by a prior (background) choice, provides insight into the dynamics driving the background-contrast effect and its attenuation by the presence of a default. In the absence of a default in the (earlier) Toronto choice, the inexpensive background contrast significantly reduces the choice probability of the higher-price Montreal hotel relative to control (b = −.30, *p* = .021). In line with our theorizing, this effect is fully attenuated in the default-present condition (b = .01, *p* = .958), such that choice probability of the higher-price Montreal hotel is significantly greater than in the default-absent condition (b = .30, *p* = .024). Similarly, in the default-absent condition, the expensive background contrast significantly increases the choice probability of the higher-price Montreal hotel relative to control (b = .87, *p* < .001). This effect is attenuated marginally in the default-present condition, in which the choice probability is lower than in the default-absent condition (b = −.24, *p* < .069) but remains significantly greater compared with control (b = .63, *p* < .001). Further results of an analysis of the focal choices conditional on the initial choice are available in Web Appendix 1.

The results of Experiment 1 show that, in line with our theorizing that accepting a default reduces the thoughtfulness of the choice, the background-contrast effect is attenuated when the initial choice was made by accepting a default. For a background-contrast effect to arise, consumers must process the background information from the first choice, make an inference from it, and apply that inference in the subsequent choice. Accepting a default reduces this inference-based choice spillover.

## Experiment 2: Sequential Choice of Different Products in a Package

Experiment 2 examines the impact of defaults on spillover between choices of different products made in sequence—a hotel and a car rental—to test our hypothesis that defaults systematically attenuate choice spillover arising in the form of preference updating. The attributes for the hotel options are identical to those in the inexpensive background-contrast condition in Experiment 1. However, Experiment 2 was designed differently from Experiment 1 on two important points so that we did not expect a background-contrast effect to emerge. First, the choices in Experiment 2 involve two different domains. They are related to each other only insofar as they are both modules in the configuration of a packaged consumption experience. Whereas inferences about the trade-off between price and quality arising from one choice are easily applied to another choice in the same domain, as in Experiment 1, mapping such inferences to a different domain, from a hotel to a car rental in Experiment 2, is more difficult and less obviously relevant. Second, the price difference associated with higher quality does not differ substantially between the two choices in Experiment 2 ($5 in the hotel choice and $7 in the car rental choice), eliminating the contrast of trade-off values that typically gives rise to a background-contrast effect. Together, these factors mitigate against the emergence of a background-contrast effect and increase opportunities for the preference signal derived from the first (hotel) choice to spill over to the focal (car rental) choice. Therefore, we expect preference updating such that a choice of the higher-price option in the first (hotel) choice when that choice does not have a default (active choice) increases the probability of participants choosing the higher-price option in the focal (car rental) choice, and attenuation of this choice spillover when the high-price hotel option is preselected as the default and accepted (passive choice).

### Method

A total of 1,845 unique residents of the United States recruited from the Prolific online research platform (www.prolific.co) completed the experiment. They provided basic demographic information, responded to an IMC, and experienced four training trials identical to Experiment 1. Participants who failed the IMC were asked to reread the instructions before continuing and were presented with the same IMC again. Participants were randomly assigned to one of three conditions (default present, default absent, and control).

Following the training trials (identical to Experiment 1), participants were introduced to a travel scenario in which they were planning a trip to Toronto, Canada and needed to book a hotel and a car rental. Each of these decisions involved choosing from two options characterized by two positively correlated attributes; quality and price (see Table 3). Participants in the experimental conditions selected the hotel first and then made the focal car rental choice. Participants in the control condition made the focal car rental choice first. In the default-present condition, the high-price hotel option was preselected, with a checkmark appearing in the selection box below that option and the statement “Recommended for you!” appearing above the selection box.

**Table 3.** Options for the Hotel and Focal Car Rental Choices (Experiment 2).

	Hotel Choice		Focal Car Rental Choice	
	Low-Price Option	High-Price Option	Low-Price Option	High-Price Option
Quality	Three-star standard	Four-star luxury	Standard sedan	Luxury sedan
Price	$134 per night	$139 per night	$42 per day	$49 per day

### Results and Discussion

We excluded data from 256 participants from analysis: 200 failed the IMC twice and 56 self-reported distractions or technical problems, leaving a sample of 1,589 (M_age_ = 35.71 years, SD_age_ = 12.49 years; 54.7% female) usable participants. Consistent with Experiment 1, examination of the nonfocal hotel choice as a function of whether a default was present does not reveal a significant effect (logit model, b = −.37, *p* = .119). In both conditions, the vast majority of participants chose the high-price hotel, which represented much better value for money (default present = 91.2%, default absent = 93.7%).

At the aggregate level, we observe that choice of the high-price car rental is significantly more likely in the default-absent condition (45.1%) compared with control (31.6%; b = .58, *p* < .001). As the vast majority chose the high-price hotel in the first choice, this difference is evidence of spillover of the choice for high price and high quality from the initial hotel choice to the focal car rental choice when no default is present. This choice spillover supports our theorizing that actively selecting a high-price, high-quality option in the initial choice facilitates preference updating that favors high-price, high-quality options and thus increases the likelihood of selecting the option with those characteristics in the subsequent choice.

In line with our theorizing, this difference in choice share is attenuated in the default-present condition (33.3%), with choice of the high-price car rental option being significantly less likely than when the default is absent (b = −.50, *p* < .001), and not significantly different from control (b = .08, *p* = .562). Because the hotel choice was calibrated to control for a possible shift in choice shares between the conditions with and without default, this pattern of results provides evidence of attenuation of choice spillover arising in the form of preference updating when a default is accepted. Nevertheless, because a small proportion of participants rejected the default in the hotel choice—in which case we expect choice spillover favoring the low-price option in the focal (car rental) choice—it is important to inspect the effects conditional on default acceptance.

As we have hypothesized, choice of the high-price car rental option in the second choice is less likely when the high-price hotel is passively chosen by accepting a default (35.8%) than when it is actively chosen in the absence of a default (47.9%; b = −.50, *p* < .001). It is interesting to note what happens when the default is rejected, as this is also an active choice that we expect to result in a choice spillover comparable to when the option is chosen in the absence of a default. Indeed, although rejecting the default in favor of the low-price hotel in the first choice is not common, we find that when it does occur, the choice share of the low-price car rental option (93.3%) is similar to when the low-price hotel is actively selected in the absence of a default (97.1%). The difference between the choice shares in these two conditions is not significant (b = −.86, *p* = .467), in support of our theorizing that rejecting a default represents an active choice, similar to choosing in the absence of a default, from which updated preferences can spill over to a subsequent choice.

The results of Experiment 2 further support our theorizing that inference-based choice spillover effects in a sequence of choices depend on whether an option is chosen when it is set as a default. In this experiment, the default was accompanied by an explicit recommendation, which may not always be the case in real-world applications. Therefore, we do not include such a recommendation in the next experiment. In addition, whereas Experiment 2 featured choices related to different experiential products in a package, the next experiment more closely resembles the subsequent field study as it involves the configuration of modules of a single product.

## Experiment 3: Sequential Choices in a Single Product Configuration

In Experiment 3, participants were presented with a scenario about purchasing a new sofa for their living room as part of a redecoration project. Each choice—selecting the construction material for the sofa frame and the fabric for the sofa upholstery—involves choosing from two options characterized by three attributes: material, durability rating, and price. Again, we are interested in the dynamic effects of the presence of a default on spillover of the same choice without a default, but in this experiment we did not calibrate a strongly dominant option in the first choice so we can also more closely study the effects associated with rejection of the default.

Our theorizing suggests that inference-based choice spillover depends on the joint effects of the nature of the initial choice, the presence or absence of a default, and the acceptance versus rejection of the default given it is present. Having not calibrated a strongly dominant option in the first choice, we have no basis on which to form a hypothesis about a difference in the aggregate choice shares between default-absent and control conditions. However, if, as we hypothesize, accepting a default attenuates preference updating but rejecting a default does not, choice of the high-price option in the focal choice will be attenuated when a default is present in the prior choice. This effect can be expected because preference updating favoring the high-price option should be attenuated by default acceptance, whereas preference spillover favoring the low-price option should not be attenuated by default rejection.

### Method

A total of 1,601 unique residents of the United States recruited from Amazon’s Mechanical Turk platform completed the experiment. Participants were randomly assigned to one of three conditions (default absent, default present, and control), provided basic demographic information, responded to an IMC, and experienced four training trials identical to the previous experiments. Participants who failed the IMC were asked to reread the instructions before continuing, and 93 who failed the IMC a second time were prevented from continuing and therefore did not complete the experiment.

Following the training trials, participants read a sofa purchase scenario involving three modules for configuration of the product. They were informed that they had already decided the sofa design—the first module—with a base price of $1,000, that they needed to choose the upholstery fabric and frame construction material—the remaining two modules—and that the cost for each option they selected would be added to the base price to determine the total price of their new sofa. The same frame and fabric options were presented across all conditions (see Table 4). The focal choice in the experiment was participants’ fabric choice. Participants in the experimental conditions selected the frame first followed by the focal fabric choice, whereas in the control condition, participants selected the fabric first. In the default condition the high-price frame option was preselected as the default, with a checkmark appearing in the box below that option.

**Table 4.** Options for Frame and Focal Fabric Choice (Experiment 3).

	Frame Choice		Focal Fabric Choice	
	Low-Price Option	High-Price Option	Low-Price Option	High-Price Option
Material	¾ inch plywood	Kiln-dried hardwood	Polyester fabric	Microfiber fabric
Durability rating	Good (3/5)	Excellent (4/5)	Good (3/5)	Excellent (4/5)
Price	$159	$259	$119	$299

### Results and Discussion

Data from 35 participants who self-reported more than one distraction or a technical problem were excluded from analysis, leaving a final sample of 1,566 (M_age_ = 38.69 years, SD_age_ = 12.40 years; 52.2% female). We first examined the nonfocal frame choice as a function of whether the default was present. As in the first two experiments, there is no significant difference in choice of the high-price frame option when it is set as a default (default present = 71.9%, default absent = 73.5%, b = −.08, *p* = .587). This may reflect a strong bimodal preference for frame options in the sample that is not shifted by the default.

Despite the lack of an immediate effect on frame choices, the default has an important effect on the subsequent focal fabric choice. Specifically, choice of the high-price option in the focal fabric choice is significantly reduced when a default is present in the first choice (68.9%) compared with when the default is absent (77.1%; b = −.42, *p* = .003). This effect conforms to our expectation that choice of the high-price option in the focal choice will be attenuated when a default is present at the high-price option in the prior choice.

Our theorizing about preference updating in the absence of a default suggests that participants selecting the high- (low-) price option in the frame choice are more (less) likely to select the high-price option in the focal fabric choice than they would have been had they not first made the frame choice. Although this theorizing does not lead to a specific hypothesis about the net effect of these opposing forces, these opposite effects on choice likelihood might in some cases cancel each other out. In accord with this possibility, we do not find a significant difference in the focal fabric choice between the default-absent (77.1%) and control (75.9%) conditions (b = .07, *p* = .631), and choice of the high-price fabric option is lower compared with control in the default-present condition (b = −.35, *p* = .012).

Although our theorizing offers an explanation for the lack of a significant difference between the default-absent and control conditions, there is another possible explanation that must be considered: that the focal choice shares reflect stable preferences. However, stable preferences cannot account for the observed reduction in choice share for the high-price option in the default-present condition. Given that the default does not affect choice share in the frame choice, a stable preferences explanation implies that the presence of default in the frame choice should also have no impact on the choice share in the focal fabric choice. By contrast, our preference updating explanation does account for the impact of the default. Specifically, our contention that accepting a default attenuates preference updating, but rejecting a default does not, implies that (1) choice of the high-price option in the focal fabric choice is reduced among those who choose a high-price frame when that frame choice is made by accepting a default, and (2) choice of the low-price option in the focal fabric choice is not reduced among those who first choose the low-price frame option when that frame choice is made by rejecting the default. Inspecting the focal choice shares conditional on frame choice offers additional insight into these hypothesized dynamic effects.

As we expected, participants’ choice of the high-price option in the focal fabric choice was significantly lower when they selected the high-price option in the frame choice by accepting the default (79.4%) compared with when they made the same frame choice in the absence of a default (85.3%; b = −.41, *p* = .034). This result is consistent with an attenuation of preference updating, but not with a stable preferences explanation. Also as we expected, participants’ choice of the low-price option in the focal frame choice was not reduced when they selected the low-price option in the frame choice by rejecting the default. Instead, the choice share for the low-price fabric option (54.3%) was significantly greater compared with when participants made the same frame choice in the absence of a default (45.7%; b = .49, *p* = .042). This result is also not consistent with a stable preferences explanation, but it does conform to our speculation that rejecting a default may boost choice spillover because it may involve an even more thoughtful choice than making the same selection in the absence of a default.

The results of Experiment 3 highlight that firms using a default with the intention of boosting sales of higher-price options might obtain a surprising outcome contrary to their intentions. In this sofa configuration scenario, the net effect of the high-price default on consumers’ final product configuration could be undesirable to a firm. The average final price of the configured sofa is lower when the high-price option is preselected as a default in the frame choice ($1,474.03) than if no default is preselected ($1,490.27; t(1017) = −2.50, *p* = .006). Rather than benefiting a firm by increasing revenue as a manager might expect, preselecting a high-quality, high-price option as a default has the potential to reduce the price customers ultimately pay for the configured product by driving some consumers to make lower-price, lower-quality selections in subsequent choices. Next, we report on a field study in which our theorizing about the moderating effect of defaults on choice spillover is tested in a more complex product configuration system and the implications of these dynamics for firm revenue are explored.

## Field Study: Sequential Consumer Choice with Defaults in an Online Car Customization System

To further validate our theorizing in the field and to illustrate the managerial relevance of the theorizing, we present a field study that was conducted in collaboration with a major car manufacturer. We observed the sequences of choices made by car buyers who used the firm’s online product customization system to configure their vehicle. Default settings were manipulated across four experimental conditions. The results shed light on how the dynamic effects of defaults revealed in the tightly controlled experiments reported above play out in a less restrained context characteristic of the product configuration environments consumers regularly encounter.

### Design of the Field Study

The field study was conducted in collaboration with a premium car manufacturer using that firm’s live online product customization system. The car customization system consisted of a series of different screens containing multiple modules, each with multiple possible options that consumers could select (for details, see Web Appendix 3). Consumers who accessed the customization system were invited to use an experimental beta version. Participating consumers were randomly assigned to one of four experimental conditions. Consumers who chose not to participate used the standard version of the customization system.^4^ The standard and experimental versions of the customization system differed only in that (unbeknownst to the consumers) different defaults were set in each of the four experimental versions, whereas the standard customization system contained no defaults.

The first step in designing the field study was the selection, from the large number of customization options available, of the modules and default options that could be manipulated. We conducted several interviews with representatives from the firm we worked with for this purpose. Although we were not permitted to change all available modules, following discussions with the firm, five modules were used to vary the defaults in the customization system: business package, rims, upholstery, steering wheels, and front seats.

We defined four treatment conditions that differed in terms of the defaults that were implemented, the extent to which high-price options were preselected as defaults, and the total number of defaults (see Table 5). The conditions were constructed in consultation with the car manufacturer such that an increasingly positive immediate effect of the default conditions on the price of the final car configuration was expected. Condition 1 included the most basic and smallest set of defaults, which were the basic type of upholstery and the business package. By contrast, Condition 4 included the most luxurious and largest set of defaults, which were high-end rims, advanced steering wheel, leather seats, electronic front seats with memory functions, and the business package. Two intermediate sets of defaults were implemented in Conditions 2 and 3.

**Table 5.** Default Settings per Experimental Condition (Field Study).

	Treatment Conditions			
Modules for Which Defaults Were Set	1 Low-Price Defaults	2	3	4 High-Price Defaults
Business package	Y	Y	N	Y
Rims 18-inch aluminum	N	Y	N	N
Rims 19-inch aluminum	N	N	Y	Y
Upholstery fabric type 1	Y	N	N	N
Upholstery fabric type 2	N	Y	N	N
Upholstery leather type 1	N	N	Y	N
Upholstery leather type 2	N	N	N	Y
Multifunction steering wheel	N	Y	Y	Y
Front seats with memory function	N	N	Y	Y

*Notes*: Y = set as default option; N = not set as default option.

The field study in the car customization system lasted 49 weeks. At the end of this period, we had obtained observations of 308 car configurations in Condition 1, 291 car configurations in Condition 2, 254 car configurations in Condition 3, 270 car configurations in Condition 4, and 7,485 car configurations in the no-defaults standard customization system.^5^ This means that 13% of all configurations were designed in an experimental system with defaults. On average, consumers configured a car priced at approximately €58,000 across all conditions.

### Model-Free Results

A key initial question is what impact the defaults have on the price of consumers’ configured cars. To gain initial insights into the prevalence of immediate effects of defaults and their subsequent effects in modules without defaults, we analyzed the prices of the configured cars as well as the prices within the modules that did/did not have a preselected default. Because much of the variation in car prices is driven by the body style (e.g., sedan, station wagon), engine, and transmission, we control for this variation by subtracting the average price level for each combination of body style, engine, and transmission from the total car price, based on the observed car configurations without defaults. Importantly, these three modules were selected on the first screen of the customization system, always before consumers encountered a default.

**Figure 1.**
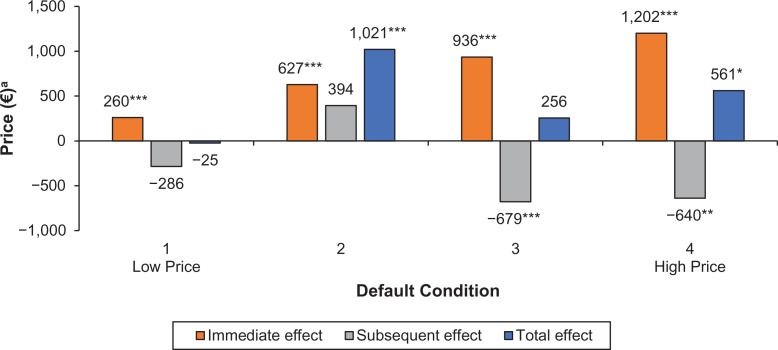
Effects of defaults on the price of the configured car. *p < .1. **p < .05. ***p < .01. ^a^Relative to no default setting. *Notes*: Labels next to the bars represent the effect in euros.

Figure 1 depicts the impact of the defaults in each of the experimental conditions on the price of the configured car relative to that in the standard customization system without defaults. The first bar represents for each condition the immediate effect of the defaults on the price paid for the corresponding modules, the second bar the subsequent effect of the defaults on the price paid for the modules that did not have defaults preselected, and the third bar the effect of the defaults on the total price. For the lowest price condition 1, the figure shows an immediate positive effect of defaults—an increase in configured price on the attributes where a default is set—of €260. The defaults also have a negative subsequent effect, decreasing configured price by €286 on the remaining attributes. Together, these opposing effects result in a net decrease in total configured price of €25.^6^ From the figure, it is clear that defaults do affect the price of the car configuration not only through an immediate effect on the focal modules as expected but also by changing consumer choices for subsequent modules. These subsequent effects are sufficiently large and different across the conditions for more positive immediate effects on price not to be revenue maximizing in all conditions when the subsequent effects are accounted for: Compare Condition 4 (which has the largest immediate effect but a negative subsequent effect) with Condition 2 (in which both the immediate and subsequent effects are positive) and it is clear that Condition 2 results in higher overall revenue despite the defaults in that condition having a smaller immediate effect than in Condition 4.

### Econometric Analysis

Although we find an impact of the sets of defaults on total price, evaluating the precise effects of each individual default on spillover is not possible in our initial analysis, as the defaults are embedded in a sequence of choices with multiple defaults in each condition. Thus, we revert to a model-based approach to infer the effects of defaults on spillover from the field data. For this purpose, we develop a model that captures the three key components of our theory: (1) there are inference-based spillover effects across choices of the various modules, (2) when a choice follows a preselected default, these choice spillover effects are attenuated, and (3) when a default is present but not chosen, these choice spillover effects are retained.

The model characterizes a sequence of choices that a consumer makes across modules that jointly make up the complete product. For each module, consumers choose between different options that can be described in terms of their features, such as price and material composition. To illustrate, a car’s module “exterior color” could have as options “metallic blue” and “black,” each with a different price and durability level as features. To study spillover effects, we model the dynamics in price sensitivity, as price is the only feature that is comparable across the different modules.^7^ Given the large variation in price levels and price ranges across modules, we rescale the prices of each module such that the price of a module option n for module m, $P_{m,n}\;$ ranges from −1 to +1 across the module options (n = 1,…, N_m_) within each module (m = 1,…, M). This ensures that there are module options that are below and above average in price.

To model consumers’ module option choices and the impact of defaults on spillover across the sequence of choices, we employ a random utility model with individual-level coefficients that capture systematic differences across consumers. Consumers are assumed to select for each module the option that provides them with the highest utility (McFadden 1986). We define a consumer’s utility for an option for a particular module as follows. For each consumer c (c = 1,…, C), module m (m = 1,…, M), and option n (n = 1,…, N_m_) with price $P_{m,n}$ , the utility that is obtained by the consumer is given by


1 $$U_{c,m,n}=\lambda_{m,n}+\alpha_{c,m}P_{m,n}+\gamma \text{I(}n={\text{Default}}_{m,c})+\varepsilon_{c,m,n}.$$

In this equation, an option’s utility is defined by three components. First, an intercept $\lambda_{m,n}$ that is specific to a particular option n within a module m that captures aspects of the option that are not directly connected to its price level $P_{m,n}$ .^8^ Second, the impact of an option’s price level is captured by the price coefficient α_c,m_, which varies across consumers to capture consumer heterogeneity and varies across modules to capture preference dynamics, as we detail next. Third, the additional utility arising from the fact that an option is set as the default is captured by the product of γ , which quantifies the change in utility, and an indicator function $\text{I(}n={\text{Default}}_{m,c})$ that is equal to 1 when option n is set as the default for module m for consumer c and 0 otherwise. If no default is specified for a given module, the value of this indicator function is also 0. Finally, the unexplained part of the consumer’s utility is captured by an error term $\varepsilon_{c,m,n}$ , which is assumed to follow an i.i.d. extreme value Type I distribution for all values of c, m and n, corresponding to the multinomial logit model.

Preference dynamics are essential to capture the process of sequential preference construction when consumers make a series of related choices in the presence of defaults. In our utility specification, these effects are formalized as a spillover of a consumer’s current choice on their utility of the available options in subsequent choices. Specifically, because price is the only common feature across attributes, a consumer’s price sensitivity shifts as a function of the option they selected in the previous choice. We account for the differential spillover effects that result from defaults affecting the preference construction process by having the update of the preference weights depend on the previous choice having been the selection of a default (passive choice) or not (active choice) and, if it was not a default, as a function of whether this active choice was made from a set that did or did not include a default.

To formally capture this effect, we model the impact of the previous module option choice on the price coefficient by introducing a spillover parameter, $\beta_{c,m}$ , that governs how the price of the chosen option influences preferences in subsequent choices. In particular, we capture shifts in the price coefficient through


2 $$\alpha_{c,m}=\alpha_{c,m-1}+\beta_{c,m}\overset{N_{m-1}}{\underset{n=1}{\sum }}P_{m-1,n}I\left(Y_{c,m-1}=n\right).$$

In this equation, the summation over the module options selects the price, $P_{m-1,n}$ , of the module option that was chosen, and $\beta_{c,m}$ captures the magnitude and direction of the change in preference weights between subsequent choices. In this specification, positive values of $\beta_{c,m}$ indicate a choice spillover effect such that a particular preference is reinforced by the consumer’s choice—for instance, when choosing a low-price option for a given module (i.e., the rescaled price $P_{m-1,n}<0$ ) results in a greater preference for lower-price options in subsequent module choices (i.e., a more negative value for $\alpha_{c,m}$ ). By contrast, negative values of $\beta_{c,m}$ indicate preference balancing across choices such that a particular preference is attenuated in the subsequent choice—for instance, when choosing a low-price option initially causes a shift in preference toward higher-price options in subsequent choices.

The impact of selecting a default or not on the spillover effect is characterized by the following, more detailed, specification of $\beta_{c,m}$ , which allows the spillover to be affected by if it is a default that is chosen and by a default being present but not chosen. These conditions, identified by $I\left(Y_{c,m-1}={\text{Default}}_{m-1}\right)$ and $I\left(Y_{c,m-1}\neq {\text{Default}}_{m-1}{\text{and Default}}_{m-1}\neq 0\right)$ , respectively, result in the following specification for the spillover:


3 $$\beta_{c,m}=\delta_{0}+\delta_{1}I\left(Y_{c,m-1}={\text{Default}}_{m-1}\right)+\delta_{2}I\left(Y_{c,m-1}\neq {\text{Default}}_{m-1}{\text{ and Default}}_{m-1}\neq 0\right).$$

The strength of the spillover that occurs in the absence of a default is characterized by the baseline shift in preference weights $\delta_{0}$ . Note that our theorizing does not provide a directional prediction for the preference updates in the absence of a default. If, as in Experiments 2 and 3, individuals learn about their preferences or are motivated to display consistency in their preference for certain types of options (e.g., for high-price, high-quality/luxury options), we would expect to observe choice spillover. This would be indicated by a positive value of $\delta_{0}$ . However, if balancing (e.g., choice of a high-price option resulting in a reduction in preference for higher-price options in subsequent choices, which may be more likely to occur when budget constraints are salient) or, as in Experiment 1, background-contrast effects dominate, $\delta_{0}$ would be negative. When multiple choice spillover effects operate, $\delta_{0}$ represents the resulting net spillover. For instance, if across a sequence of choices both preference updating and balancing occur, the sign of $\delta_{0}$ will depend on the relative magnitude of these two choice spillover effects.

The other two components of Equation 3 capture differences in the spillover process that result when a default is present. If, as we propose, the choice to accept the default is more passive than a choice made in the absence of a default, this should attenuate inference-based choice spillover. Inference-based spillover effects thus drive $\delta_{0}$ , which captures the baseline spillover effect, and $\delta_{1},$ which captures the incremental change in spillover when the default is accepted, in opposite directions. Because the total configured price up to the current point in the customization process is highly salient in the configurator interface, we expect the baseline spillover effect in the field data to include a balancing component that results from the consumer’s budget constraint. Because budget-based balancing is a less inference-based choice spillover, the balancing component in the net spillover will be attenuated less by default acceptance compared with the positive, inference-based, preference-updating component. Under such circumstances, the incremental spillover effect of accepting a default, $\delta_{1},$ is expected to be negative and can be even larger in magnitude than $\delta_{0}\;$ because the positive inference-based components of the spillover effect will be more strongly attenuated than the negative (non-inference-based) balancing component. When a default is present but rejected, we also allow spillover to differ from baseline, as captured by $\delta_{2}$ , because even though default rejection is also an active choice, it may involve differences in choice processing either by eliciting even more thoughtful choice or via perception of the default as an implicit recommendation. In the former case, spillover is shifted toward the chosen option and away from the defaulted option, and in the latter case, it is shifted toward the defaulted option and away from the chosen option.

When estimating models with dynamic preferences, an important consideration is that one must be cautious before concluding that changes in estimated preferences truly reflect a changing data-generating process and not an increasingly more accurate assessment of a stable preference, because more data become available when observing subsequent choices. In particular, when the researcher assumes homogeneous preferences at the start of the process, this results in spurious learning of the consumer-level preferences from behavior (Shin, Misra, and Horsky 2012). This stems from the fact that the estimated preference weight for a specific feature for a consumer who systematically chooses module options that score high on that specific feature will increase as more data become available. This, however, is not because this person learned that they prefer high levels of this module feature but because the researcher learned this. This is similar to the need to control for systematic differences in preferences to properly measure spreading of preferences using the free-choice paradigm (Chen and Risen 2010).

To prevent spurious learning effects, we include a consumer-level random parameter $\alpha_{c,1}$ to capture consumer heterogeneity at the start of the choice process. These random parameters are assumed to follow a normal distribution. The mean of the distribution is fixed at zero, as the level of α is not identified when module option intercepts $\left(\lambda_{m,n}\right)$ are included and there is no variation in the option features across individuals. The variance is set at σ_α_^2^. We executed a simulation study to verify the validity of this approach. The simulation study was based on the estimated model parameters from the field study and showed that (1) we are able to recover the correct spillover effects when heterogeneity is accounted for, (2) not accounting for initial preference heterogeneity biases the estimated spillover effects as expected, and (3) when there are no spillover effects, the approach does not infer spurious spillover effects. Details of the simulation study are provided in the Appendix.

Another aspect of the data that we control for in the econometric model is that the diagnosticity of the features, in terms of spillover effects, might vary across modules. This is especially relevant for our common feature, which is price. To illustrate such differences in diagnosticity across modules, consider a price difference of €200 between two engine options. Given that engines are generally the most expensive module of a car, consumers’ responses to a €200 price difference between engine types might be small and less diagnostic about their underlying module option preferences than a €100 price difference for a (much less expensive) steering wheel. To allow for such differences across modules, we include a module-specific scaling factor ( $\theta_{m})\;$ that rescales the observed price levels to values that are comparable across modules (i.e., throughout our model specification we use $\theta_{m}P_{m,n}$ instead of $P_{m,n}$ ).

We perform model estimation using simulated maximum likelihood, where the simulation is used to account for consumer heterogeneity in their initial level of price sensitivity. Table 6 presents the results for the key parameters representing the immediate and dynamic effects of defaults on consumer preference, as well as the baseline dynamic choice spillover effect. We find that, as expected, there is a strong and positive immediate default effect on the probability that a specific module option is chosen ( γ = 1.023, *p* < .001).

**Table 6.** Effects of Defaults on Consumer Utility.

	Estimate	SE	*p*-Value
Immediate default effect (γ)	1.023	.037	<.001
Choice spillover effects			
Spillover effect from choice when no default is present ( $\delta_{0})$	.083	.010	<.001
Incremental spillover from default choice ( $\delta_{1})$	−.307	.053	<.001
Incremental spillover from not choosing the default when it is present $\left(\delta_{2}\right)$	−.116	.040	.002

We also find a significant choice spillover effect of a consumer’s current option choice on their price sensitivity in subsequent choices. The baseline spillover effect of choosing a more (less) expensive option when no default is set is that this choice reduces (increases) the consumer’s price sensitivity in later choices, in line with the predictions based on self-perception and cognitive dissonance reduction. This choice spillover effect is reflected by the positive value for $\delta_{0}$ (.083, *p* < .001). We interpret this positive choice spillover to indicate that preference updating dominates, such that any opposing contribution from balancing must be of lesser magnitude.

The presence of a default changes the spillover effect. When a consumer chooses an option that is preselected as the default, the choice spillover effect of making an active choice is attenuated. Consumers’ preferences for the options of the next module no longer shift toward the chosen option (i.e., toward the default). The effect of selecting the default option on spillover to subsequent choices is reflected in the negative value of $\delta_{1}$ (−.307, *p* < .001), which, together with the baseline spillover effect, results in a net negative choice spillover effect ( $\delta_{0}+\delta_{1}$ = −.224, *p* < .001) compared with when no default is present. When a high- (low-) price option that is preselected as a default is chosen, preference for high- (low-) price options in the subsequent choice are weakened. We interpret this negative effect as indicating that the balancing component comes to dominate when preference updating is attenuated by default acceptance. We surmise that this balancing effect is driven at least in part by a budget constraint, which could be especially resistant to attenuation when the budget constraint is made salient by having the running total price visible in the configuration interface (which it always was in the field study). Balancing on a budget constraint is expected to be less sensitive to attenuation by default acceptance because a budget constraint remains constant across modules regardless of the choice process.

When a default option is present, but the consumer deviates from this default and instead (actively) chooses a different option, the effect of the default on spillover is also in the opposite direction of the baseline spillover effect, but much less strongly so than when the default option is chosen ( $\delta_{2\;}$ = −.116, *p* = .002). This finding is consistent with the notion of a default serving as an implicit recommendation, even if the consumer does not selected it. In this case, the net impact of the current choice on a consumer’s preferences as revealed in subsequent choices is statistically indistinguishable from zero $\left(\delta_{0}+\delta_{2}\approx 0\right)$ .

## General Discussion

This research examined the impact of defaults in a sequential choice context using experimental data from three studies and field data from customers of an automobile manufacturer who configured their cars online. We find that defaults have a systematic effect on choice spillover in sequential choice processes and that this effect depends on whether a default has been accepted or rejected. Our findings have both theoretical and practical implications.

### Practical Implications

Our studies reveal that the presence of a default in one choice of a sequential choice process has an impact on subsequent choices. From a managerial perspective, this finding has potential consequences for the use and calibration of default settings and their impact on consumer purchase behavior in sequential choice settings. Gaining a detailed understanding of these consequences is important and nontrivial, given the complex pattern of immediate and spillover effects we observed in the field study. Therefore, we now demonstrate how model-based simulations can be used to gauge the immediate effects of defaults as well as their effects on spillover of consumers’ constructed preferences to subsequent choices (e.g., Dew and Ansari 2018). The key to understanding the impact of a default option on consumer behavior lies in an analysis of the combined effect of the immediate effects of defaults and their effects on subsequent choices. To illustrate the total impact of defaults in the car customization system from a managerial perspective, we focus on the shift in consumer spending. This focus also allows for a direct comparison of the immediate and subsequent effects.

**Figure 2.**
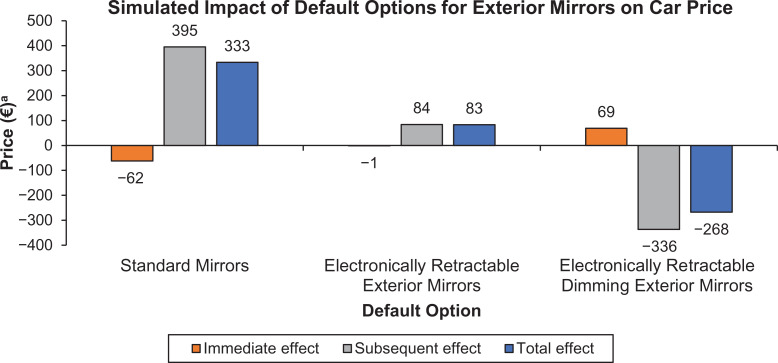
Illustration of offsetting spillover effects. ^a^Relative to no default setting.

For illustrative purposes, we study the possible default settings for the exterior mirrors module in the car customization system. Figure 2 shows for each option of this module the predicted immediate, subsequent, and total effects on the price of the configured car when an option in this module is preselected as a default. For the lowest-price option, the immediate effect is a reduction in the price of the selected mirrors of €62, the subsequent effect is an increase of €395 in the price of the car, and these two opposing effects result in a predicted overall increase of €333 in the average price of the configured car. By contrast, when the highest-price option is preselected as the default, the immediate effect is an increase of €68 in the price of the selected mirrors, the subsequent effect is a €336 reduction in price, and the resulting total effect is a reduction in the average price of the configured car by €268. Counterintuitively, in this illustrative example, preselecting the highest-price option as the default is detrimental to the firm, in spite of a gain in revenues on the exterior mirrors module itself. Meanwhile, the simulation predicts that setting the lowest-price option as the default benefits the firm.

An important feature of the exterior mirrors module is that both the lowest-price and the highest-price options are rather attractive (chosen in about 55% and 30% of the configured cars). This helps explain the strong subsequent effects of the defaults. Consider the 55% of customers who choose the lowest-price option. In the absence of a default, choosing their preferred low-price option results in preference updating in favor of low-price options in subsequent choices. But when this same choice is made by accepting a default, that choice spillover is attenuated and preference balancing remains. Thus, the spillover to subsequent choices favors higher-price options to a greater degree than if these customers had actively made the same low-price mirror choice in the absence of a default.

When performing the same analysis for all possible defaults in the car customization system that we analyzed, we find that for 111 options (67.3% of all options) the subsequent effect is opposite to the immediate effect on the price of the configured car, so focusing only on the immediate effect would overestimate the impact of a default on firm revenues. For 52 of these options (31.5% of all options) the subsequent effect on price is not only opposite but also larger in magnitude. For 38 of these options, all of which are low-price options set as defaults, the immediate effect on price is negative and the total effect is positive, while for 14 options across 9 modules we find that a positive immediate effect of setting the option as a default would result in a negative total effect on revenues.

Managers who intend to use defaults to increase total revenues or profits thus need to look beyond the focal module when setting default levels in a context where consumers go through a sequence of related choices. Defaults that attenuate spillover of preference for higher-price options will be less effective than the immediate effect would suggest and may even have a total impact opposite to the one anticipated when only the immediate effect would be accounted for. Setting a high-price option as the default brings with it the risk of backfiring and hurting total revenues. Another important implication of our findings, which managers may also find surprising, is that there are circumstances under which setting a low-price option as the default in one module could increase total revenues by boosting choice of higher-price options in subsequent modules.

### Theoretical Implications

From a theoretical perspective, our research provides new insights into the dynamic impact of defaults on consumer decision making. While there is much evidence in the literature concerning the immediate effects of defaults on consumer choice (Brown and Krishna 2004; Johnson and Goldstein 2003; Smith, Goldstein and Johnson 2013), to the best of our knowledge we are the first to investigate dynamic effects of defaults on consumer preferences in subsequent choices in sequential choice processes. We propose that accepting a default is a more passive choice than both rejecting a default or making a choice in the absence of a default. In a series of experiments and a field study, we showed that choice spillover effects are indeed influenced by the presence of a default, as we predicted. Analysis of individual choice sequences reveals that inference-based choice spillover effects are attenuated when the default is accepted.

The effect of rejecting a default on choice spillover is more variable and is worthy of future research. Rejecting the default has the potential to increase preference updating, as we find in Experiment 3, possibly because consumers engage additional mental resources to consider why the default should be rejected. In Experiment 2 and in the field study, however, rejecting the default results in spillover effects similar to or smaller than those that arise when making that same choice in the absence of a default, possibly due to a recommendation effect.

In the field study using a sequence of choices required to configure a complete car online, we relied on an econometric model of sequential choice behavior. This model allowed for preferences for features of the product to be constructed during the choice process. Estimation results provide evidence that later preferences (as revealed by subsequent choices) are predictably influenced by consumers’ earlier choices (i.e., choice spillover, dynamic preference construal). Importantly, these dynamics in consumer preferences were also modulated by the presence of defaults, resulting in effects of defaults on spillover to subsequent choices. We anticipated that, in the field data, preference reinforcement through consumer self-perception and cognitive dissonance reduction results in choice spillover across modules that is reduced by the presence of a default. Our findings support this prediction. When consumers chose an option that was set as the default option, we find that the baseline (default-absent) choice spillover effect of such choices reverses. Instead of an increase in consumers’ preferences in the next choice in line with the features of the chosen default, there is net negative effect indicative of balancing. This switch in the direction of the spillover effect due to a default suggests that there are multiple spillover effects at play that differ in the degree to which they are inference-based (preference updating vs. balancing) and that are differentially attenuated when a choice is made through default acceptance.

## Conclusion

The topic of this research—sequential decisions with defaults—is relevant in many markets where consumers make a sequence of interrelated choices, such as when configuring products (e.g., holiday packages, financial services) or when selecting multiple options from a menu (e.g., a multicourse restaurant meal). Although we tested the proposed effects of defaults on choice spillover in tightly controlled experiments and in field data that showed practical relevance by studying important and impactful consumer decisions in the context of a car customization tool, it would be interesting to examine whether these findings hold in other types of markets where spillover of constructed preferences may occur. In particular, it could be interesting to see under which conditions subsequent choices are sufficiently related to versus different from each other for defaults to have an influence on spillover (e.g., Priester, Dholakia, and Flemming 2004). For example, multiple decisions within the same product category in a store could well be related enough for defaults to moderate choice spillover effects (e.g., buying multiple fashion items), whereas decisions made in different product categories in the same store (e.g., buying fashion items and consumer electronics) may constitute different decision episodes that are not cognitively connected by consumers (Dhar and Simonson 1999).

Another line of research that would be worthwhile to pursue in future work is to study in greater detail the (possibly conflicting) underlying processes in consumer decision making that determine the strength and direction of spillover effects and how these processes are affected by the presence of defaults. In this research, we have established that defaults have systematic effects on choice spillover and that these effects are dynamic in nature. However, these dynamic effects may not always apply to balancing and may be moderated by various factors. It would be worthwhile to examine in greater depth the proposed process accounts of consumer inference and active versus passive choice. For example, the salience of a budget constraint (or a weight loss goal, in the case of nutritional features of products) may moderate effects of defaults on preference balancing. In addition, depending on the level of involvement in the decision, consumers may be more or less sensitized to the fact that their decision is partially determined by the presence of a default. It is likely that depending on their awareness of this influence, the effect of the default on choice spillover may also vary in strength. Similarly, the type of default, a simple checkmark or a more descriptive label (e.g., “Selected for You”) may result in different attention levels and inferences that affect the impact of the default on spillover effects. Consumers’ beliefs about the firm’s motives in providing defaults (e.g., profit maximization, consumer satisfaction) may also change their inferences. We hope that the current work will serve as a foundation for such further investigations into the dynamic effects of defaults.

## Supplemental Material

Supplemental Material, Web_Appendix_final_PDF - Preference Dynamics in Sequential Consumer Choice with Defaults Supplemental Material, Web_Appendix_final_PDF for Preference Dynamics in Sequential Consumer Choice with Defaults by Bas Donkers, Benedict G.C. Dellaert, Rory M. Waisman and Gerald Häubl in Journal of Marketing Research

## Appendix: Simulation Study on Heterogeneity Correction in Field Study

The fact that choices and preferences are confounded makes the analysis of the impact of a choice on subsequent choices challenging. In the psychological literature, this has been identified by Chen and Risen (2010), while Shin, Misra, and Horsky (2012) illustrated its consequences from a marketing modeling perspective.

This appendix reports the results of a simulation study that shows that controlling for initial preference heterogeneity through a random-effects model can solve this problem. This mimics the premeasurement proposed as a solution by Risen and Chen (2010) through a latent estimate of the (unobserved) initial preferences.

The simulation study relies on a scenario with 20 attributes, each with three levels. The levels have equal baseline utilities but differ in their price levels. Each attribute has one level priced at 1, one level priced at 0, and one level priced at −1, consistent with the normalization of prices used in the main model. Half of the simulated sample has no defaults, and the other half receives a default for attributes 10 and 15 in the sequence. The level that is set as default for each attribute is randomly selected from the three levels of that attribute. The specification of preference heterogeneity and preference dynamics is based on the model developed for the field study. Finally, customers’ choice behavior is simulated based on the specified econometric model and the parameter estimates we obtained.

To analyze the accuracy of the proposed approach, we estimate the full model, as well as a model that does not account for initial preference heterogeneity, for comparison purposes. The results are reported in Table A1. In addition, we simulate data where there is preference heterogeneity, but no preference updating (i.e., all δ parameters as reported in the article are zero). The latter simulation enables us to test whether the proposed approach spuriously picks up choice spillover even though it is not present in the data.

The results from the simulation study show that the proposed approach that controls for initial preference heterogeneity is indeed able to retrieve the correct parameter values—that is, the results are all very close to the parameter estimates obtained from the field data that were used to generate the simulations. The results also show that when the heterogeneity in initial preferences is not accounted for in the model, this leads to an overestimation of the baseline spillover effect, with δ_0_ = .277, which falsely suggests much larger baseline spillover effects than those that underlie the simulated data (δ_0_ = .083). Thus, there is a clear need to account for heterogeneity as we do in the proposed approach. The final column in Table A1 shows that when there is no preference updating, this is also correctly inferred by the proposed approach, and no spurious spillover is identified, as all preference update parameters are estimated to be virtually zero, though δ_0_ is statistically significant.

**Table A1.** Simulation Study Results.

	True (Input) Value	Our Approach	Approach Without Correction for Preference Heterogeneity	Scenario Without Preference Updating (δ = 0)
δ_0_	.083	.084	.277	.006
		(.002)	(.001)	(.002)
δ_1_	−.307	−.304	−.326	−.002
		(.006)	(.004)	(.006)
δ_2_	−.116	−.104	−.103	−.004
		(.006)	(.004)	(.005)

*Notes*: The table shows mean parameter estimates (with standard deviations in parentheses).

## Notes

1 This end-of-study procedure was used in all three experiments. For details of the distraction self-report and exclusion criteria that had been established a priori and were applied in all experiments, see Web Appendix 2.

2 The training choices were (purposefully) not informative with respect to our theorizing because they were identical for all participants and designed to ensure exposure to all key features of the choice interface.

3 The choice shares observed in these pretests guided our sample size target of 500 per condition.

4 This aspect of the experimental approach was imposed by our industry partner. It would have been preferable to use random assignment between control and the cluster of treatment conditions. It is important to note, however, that this self-selection only affected whether consumers entered into any of the experimental conditions with defaults (vs. the control condition). They were assigned randomly to one of the four treatment conditions. Thus, self-selection is a potential contaminant of comparisons to control, but not of any differences among the four treatment conditions.

5 Although the number of customers (and thus the overall attrition rate) was approximately equal across experimental conditions, we cannot rule out the possibility that attrition might have been affected by the different default settings in each of the conditions.

6 The €1 difference stems from rounding.

7 The model can be modified to any setting in which consumers make a series of choices and where these choices have at least one common preference attribute (e.g., calories in a multicourse restaurant meal, physical intensity in a sequence of recreational activities).

8 The first module option (n = 1) is used as a reference category so $\lambda_{m,1}=0$ that for all modules.
