# Supplementary material for: Preference Dynamics in Sequential Consumer Choice with Defaults
Source: J Mark Res. 2020 Oct 14;57(6):1096–112. doi: 10.1177/0022243720956642 (PMC13038157; doi:10.1177/0022243720956642)
Supplement: Supplemental Material, Web_Appendix_final_PDF - Preference Dynamics in Sequential Consumer Choice with Defaults [file Web_Appendix_final_PDF.pdf]

WEB APPENDIX

Table of Contents

Web Appendix 1. Conditional Analysis for Experiment 1 2

Web Appendix 2. Data Exclusion Criteria 4

Web Appendix 3. Model Identification and Module Options In the Field Study.............. 8

WEB APPENDIX 1. CONDITIONAL ANALYSIS FOR EXPERIMENT 1

To gain additional insight in the results of Experiment 1, we break down the focal (Montreal) choice shares for the high-quality hotel by whether a default was present *and* whether a participant chose the background-favored hotel in the (first) Toronto choice. We report coefficients and p-values stemming from a logit model for the choice of the high-price high-quality Montreal hotel, as a function of the three-way interaction between the participants initial choice, the background contrast (expensive vs inexpensive) and the presence of a default, while controlling for main effects of the background contrast and default presence.

For the default-absent inexpensive background-contrast condition, participants who chose the background-favored high-quality (Toronto) hotel are also more inclined to choose the high-quality hotel in the focal (Montreal) hotel choice (choice share = 47.7%) compared to those who chose the low-quality hotel (choice share = 20.0%; b = 1.60, $p < .001$). This reflects the combined impact of the participants' low inherent price sensitivity and the spillover effect from the background contrast. When the background-favored hotel was preselected as a default, the spillover effect was attenuated for participants choosing the high-quality hotel (share = 40.7%; b = .29, $p = .037$). Similarly, participants choosing the background-favored low-quality Toronto hotel in the expensive background-contrast condition were less likely to choose the high-quality hotel in the focal (Montreal) hotel choice (= 56.1%) compared to those who chose the high-quality Toronto hotel (share = 98.2%; b = -3.09, $p < .001$). Attenuation of the background-contrast effect for participants accepting the default was weaker for the expensive background contrast (share = 61.4%; b = -.22, $p = .117$). Spillover effects resulting from choosing the non-favored hotel in the initial (Toronto) choice were not affected by the presence of a default, that

then had to be rejected, neither in the inexpensive background-contrast condition (default-absent share= 20.0%; default-present share = 12.2%%; b = .59, *p* = .343), nor in the expensive background-contrast condition (share default absent = 98.2%; share default present = 97.2%; b = .43, *p* = .726).

WEB APPENDIX 2. DATA EXCLUSION CRITERIA

Inattentive responding is an increasing concern with online recruitment of experimental participants. In the summer of 2018, social science researchers observed unusual decreases in data quality and increased inattentive responding when using Amazon's Mechanical Turk to recruit participants (Moss and Litman 2018). Empirical investigation confirmed that this observation was valid (Chmielewski and Kucker 2020), revealed that much of the problem could be traced to MTurk accounts that were bypassing location restrictions by using proxy servers (Dennis, Goodson, and Pearson 2018, Moss and Litman 2018), that these accounts were being operated by humans (as opposed to programmed bots; Moss and Litman 2018), and that the people operating the offending accounts were of Indian cultural background (Litman et al. 2018, Moss and Litman 2018). Operators of such accounts were labeled "farmers" due their use of server farms to mask their true location (Moss and Litman 2018). It was also observed that open ended responses from farmers tended not to address the prompt at rates sharply greater than non-farmers, with many common responses such as "NICE" and "good study" appearing across many farmer accounts regardless of the prompt.

Confronted with the increasing concerns about inattentive responding in online samples we established, a priori, a set of protocols to prevent our pretests and experiments from being compromised by inattentive participants. We adopted the recommendation of Hauser, Paolacci, and Chandler (2019) to use multiple methods in conjunction to monitor for inattentive responding in order to prevent or exclude suspect data.

The first tools we implemented were prescreening capabilities to prevent participation from IP addresses known to be associated with server farms and participation from IP addresses

already associated with a prior participant. Second, we used an instructional manipulation check (IMC; Oppenheimer et al. 2009) to screen for inattentive responding prior to assigning participants to an experimental condition. In Experiments 1 and 3 the IMC was used to prevent inattentive MTurk workers from participating ex ante. In Experiment 2 the policies of the Prolific recruitment platform prevented us from implementing this procedure in the same way. In this case, participants who failed the IMC twice were allowed to continue through the experimental protocol and their data was removed during ex post data cleaning, yet *prior* to any review of experimental data and analysis.

The third tool we utilized to monitor for inattentive participants was a self-report distraction checklist (Figure WA1) completed by participants at the end of each experiment. Participants reporting a technical problem or a distraction from something else in their environment (the last two items on the checklist) were asked to briefly describe the technical problem or distraction in an open-ended response. In prior, unrelated experiments conducted by two of the authors, it has been observed that participants self-reporting more than one distraction including either of these last two items tend to provide open-ended responses that either do not address the prompt (e.g., “NICE” or “good study”) or deny the existence of the distraction or problem they have reported (e.g. “NO”, “no problem”), both of which are indicators of inattentive responding.

We committed, a priori, to excluding data from participants that reported having experienced more than one source of distraction. We also committed to reviewing the open-ended responses of those participants who self-reported only one distraction using one of the last two items on the self-report checklist, and to exclude their data from analysis when they reported a distraction that was clearly more substantial than any other distraction on the checklist (e.g. had

to change my child’s diaper), when they reported a technical problem that could have interfered with the experimental protocol (e.g., my browser froze up for a while), or when their open-ended response did not address the prompt or denied the existence of the distraction or technical problem. We acknowledge that the seriousness of a distraction or technical problem is a subjective judgment. However, these exclusion criteria, including review of open-ended responses, were applied before any analysis or inspection of participants’ other responses to the core experimental measures so as to ensure that exclusion decisions could not be, even implicitly, influenced by knowledge of the participants’ responses to the experimental measures.

Inspection of the exclusion rates across conditions in each experiment did not reveal evidence of differences (Exp. 1 $\chi^2(4) = 5.88$, $p = 0.208$; Exp. 2 $\chi^2(2) < 1$; Exp. 3 $\chi^2(2) < 1$). Comparison of the self-reported gender of excluded to non-excluded participants revealed no evidence of differences (Exp. 1 $\chi^2(1) = 1.24$, $p = 0.265$; Exp. 2 $\chi^2(1) < 1$; Exp. 3 $\chi^2(1) < 1$). Likewise, comparison of the self-reported language used in daily life of excluded to non-excluded participants revealed no evidence of differences ($\chi^2(1) < 1$). Comparison of the self-reported age of excluded to non-excluded participants revealed that excluded participants were, on average, younger than non-excluded participants in both experiments conducted using MTurk participants (Exp 1. $M_{excluded} = 34.98$, $SD_{excluded} = 11.74$; $M_{included} = 38.22$, $SD_{included} = 12.39$; $t=2.784$, $p=.006$; Exp 3. $M_{excluded} = 34.46$, $SD_{excluded} = 10.24$; $M_{included} = 38.69$, $SD_{included} = 12.4$; $t=2.408$, $p=.021$) but not in Experiment 2, in which participants were recruited using the Prolific platform ($M_{excluded} = 33.34$, $SD_{excluded} = 11.88$; $M_{included} = 35.71$, $SD_{included} = 12.49$; $t=1.47$, $p=.148$).

Figure WA1: Self-Report Distraction Checklist

Thank you for your attention and effort.
You are almost finished the study.

Following is a list of things that sometimes occur during online studies. Please indicate whether or not you experienced any of these things at any time during the study.

We collect this information to ensure our data is valid for analysis. Your answers to this question will not impact your compensation for this study.

	YES	NO
Watched TV	○	○
Listened to music or radio	○	○
Used social media	○	○
Used a phone to talk or text	○	○
Talked to someone	○	○
Used other software on the computer	○	○
Experienced technical problem or software malfunction	○	○
Was distracted by something else in my environment	○	○

CONTINUE

You indicated that you have experienced a technical problem or software malfunction. Please use the space below to briefly describe the problem or malfunction:

CONTINUE

You indicated that you have experienced some type of distraction during the study. Please use the space below to briefly describe the distraction:

CONTINUE

References

Chmielewski, Michael, and Sarah C. Kucker (2020), "An MTurk Crisis? Shifts in Data Quality and the Impact on Study Results," *Social Psychological and Personality Science,* 11 (4), 464–473.

Dennis, Sean A., Brian M. Goodson, and Chris Pearson (2018), “MTurk Workers’ Use of Low-Cost “Virtual Private Servers” to Circumvent Screening Methods: A Research Note,” Working Paper.

Hauser, David, Gabriele Paolacci, and Jesse Chandler (2019), “Common Concerns with MTurk as a Participant Pool: Evidence and Solutions," In Kardes, Frank R., Paul M. Herr, and Norbert Schwarz, eds. *Handbook of Research Methods in Consumer Psychology*. Routledge.

Moss, Aaron J., and Leib Litman (2018), "After the Bot Scare: Understanding What’s Been Happening with Data Collection on MTurk and How to Stop it," blog post.

WEB APPENDIX 3. MODEL IDENTIFICATION AND MODULE OPTIONS IN THE FIELD STUDY

The proposed model allows for the construction of consumers' preferences while going through the series of choices needed to completely configure a car. The data structure used in the estimation assumes that consumers selected options in the order they were presented to them in the customization system. The data provided by our industry partner does not specify the frequency or nature of deviations from this order. However, such deviations are known to be rare but possible in practice. The impact of such deviations from the assumed sequence of choices induces noise into the dynamic model that we propose, which creates a downward bias in the spillover effects we find. Hence, in the (unlikely) case that consumers strongly deviate from the inherent ordering in the customization system, our estimates provide a conservative estimate of the actual spillover effects and their modulation by defaults.

Since we are analyzing consumer response based on price, in the data we collapsed all module options that have the same price into one option. For the color of the car, for example, this leads to a reduction from 18 different colors, to 3 price options for the differentially priced colors in the color module. We also removed 35 extremely infrequently purchased modules from the analysis (for example, an electric folding table), and if for a given module one option was purchased very infrequently, this option was merged with the one that was closest in price. The analyses were performed on a total of 58 remaining modules that jointly had a total of 165 module options, including an "absent" option where applicable (from an original total of 290 options). We provide an overview of these modules below.

Description of Options by Module

1	Engine Type 1
1	Engine Type 2
1	Engine Type 3
1	Engine Type 4
1	Engine Type 5
1	Engine Type 6
1	Engine Type 7
1	Engine Type 8
1	Engine Type 9
2	Body Style Sedan
2	Body Style Coupe
2	Body Style Station wagon
3	Transmission Type Regular
3	Transmission Type Automatic
3	Transmission Type Continuously Variable Transmission
4	No Business Package
4	Business Package
4	Business Package Plus
4	Business Package Advanced
5	No S line Exterior Package
5	S line Exterior Package
6	No Optic Package Black
6	Optic Package Black
7	No Optic Package Aluminum
7	Optic Package Aluminum
8	No S line Sports Suspension
8	S line Sports Suspension
9	No S line Sport Package Plus
9	S line Sport Package Plus
10	No Exclusive Line Package
10	Exclusive Line Package
11	Standard Rims
11	Alum.-Rims 7.5Jx16” 6-Double-crossings-Design
11	Alum.-Rims 8Jx18” 10-crossings-Design
11	Alum.-Rims 8Jx17” 7-crossings-Star-Design
11	Alum.-Rims 8Jx18” 5-crossing-V-Design
11	Alum.-Rims Brand exclusive\8Jx18” 10-crossing-Design
11	Alum.-Rims Brand exclusive\8 5Jx19” 5-Segmentcrossing-Design
12	No Bluetooth Phone with Wireless Remote
12	Bluetooth Phone with Wireless Remote
13	Default Front Seats
13	Sport Front Seats
13	S Sport Seat
14	Default Upholstery
14	Upholstery Fabric Type 1
14	Upholstery Fabric Type 2
14	Upholstery Alcantara Leather Combination
14	Upholstery Alcantara Leather Combination Plus
14	Upholstery Leather Type 1
14	Upholstery Leather Type 2
15	Regular Exterior Mirror
15	Exterior Mirror Electrically Retractable
15	Exterior Mirror Electrically Retractable and Dimmable
16	No Leather Cover for Arm Rest in the Door
16	Leather Cover for Arm Rest in the Door
17	Regular Car Glass
17	Tinted Car Glass
17	Tinted and Insulating Car Glass
17	Insulating Car Glass
18	Regular Lights
18	Xenon Plus Lights
19	No Adaptive Lights
19	Adaptive Lights
20	No Headlight Assistant
20	Headlight Assistant
21	No Sunroof
21	Glass Sunroof
21	Electric Solar Sunroof
22	No Hitch
22	Hitch
23	Regular Inside Mirror
23	Inside Mirror Dims Out Automatically
24	Decor Type 1
24	Decor Type 2
24	Decor Type 3
24	Decor Type 4
24	Decor Type 5
24	Decor Type 6
25	Regular Roof Liner
25	Roof Liner Fabric Black
26	No Storage Package Type 1
26	Storage Package Type 1
27	No Storage Package Type 2
27	Storage Package Type 2
28	No Blinds for Back Window
28	Blinds for Back Window (Manual)
28	Blinds for Back Window (Electronic)
29	No Auxiliary Heating System with Remote
29	Auxiliary Heating System with Remote
30	Regular Air Conditioning
30	Air Conditioning Comfort Plus
31	No Garage Door Opener
31	Garage Door Opener
32	Regular Lights
32	Lights Package
33	Regular Steering Wheel
33	Multi-Funct Leather Steering Wheel

4-Crossings Design
33 Multi-Funct Leather Steering Wheel
4-Crossings Design with Paddle Shift
33 Multi-Funct Sports Leather Steering Wheel
3-Crossings Design
33 Multi-Funct Leather Steering Wheel
4-Crossings Design Heated
33 Multi-Funct Sports Leather Steering Wheel
3-Crossings Design with Paddle Shift
33 Multi-Funct Leather Steering Wheel
4-Crossings Design with Paddle Shift Heated
34 No Exclusive Gearshift Lever Wood
34 Exclusive Gearshift Lever Wood
35 Regular Steering Wheel
35 Electronically Adjustable Steering Wheel
36 No Electronic Lumbar Support for Front Seats
36 Electronic Lumbar Support for Front Seats
37 No Central Arm Rest for Front Seats
37 Central Arm Rest for Front Seats
38 Regular Front Seats
38 Electronic Front Seats
38 Driver Seat with Memory Function and
Electronic Front Seats
38 Memory Function for Front Seats
39 No Seat Heating
39 Seat Heating Front Seats
39 Seat Heating Front Seats and Outer Back Seats
40 No Retractable Backseat Rest
40 Retractable Backseat Rest
41 Regular Suspension
41 Sport Suspension
41 Adaptive Air Suspension
42 No Side or Lane Assist System
42 Lane Assist System
42 Side Assist System
42 Side and Lane Assist System
43 No Parking System
43 Parking System
43 Parking System Plus
43 Parking System Advanced
44 No Speed or Cruise Control
44 Speed Regulation System
44 Adaptive Cruise Control
45 Regular Trunk Lid
45 Automatic Trunk Lid
46 No Driver Information System
46 Driver Information System
46 Driver Information System with Color Display
47 Regular Door Lock
47 Power-Assisted Door Lock
48 No Alarm System
48 Theft Warning/Alarm System
49 No Side Airbags Back Seats
49 Side Airbags Back Seats
50 Regular Key
50 Comfort Key
51 No Navigation System
51 Navigation System
51 Navigation System Plus
52 Regular Radio
52 MMI Radio Plus
53 No Phone Setup
53 Phone Setup (Preparation for Bluetooth Phone)
53 Bluetooth Phone
54 Regular Sound System
54 DSP Sound System
54 BOSE Surround Sound System
55 No Rear Seat Entertainment Setup (Preparation)
55 Rear Seat Entertainment Setup (Preparation)
56 No CD-Player/Changer for MMI Radio or MMI Radio Plus
56 CD-Player/Changer for MMI Radio or MMI Radio Plus
57 No Brand Music Interface
57 Brand Music interface
58 No TV and Digital Radio Reception
58 TV and Digital Radio Reception
